# Deconvolving cell-type-specific gene expression profiles from bulk RNA-seq samples

**DOI:** 10.1371/journal.pcbi.1014101

**Published:** 2026-03-26

**Authors:** Sichen Zhu, Zhengqi Wang, Kevin D. Bunting, Peng Qiu

**Affiliations:** 1 Department of Biomedical Engineering, Georgia Institute of Technology and Emory University, Atlanta, Georgia, United States of America; 2 Department of Pediatrics, Division of Hematology and Oncology, Emory University, Atlanta, Georgia, United States of America; 3 Aflac Cancer and Blood Disorders Center, Children’s Healthcare of Atlanta, School of Medicine, Emory University, Atlanta, Georgia, United States of America; Chinese Academy of Science, CHINA

## Abstract

Bulk RNA sequencing (bulk RNA-seq) and single-cell RNA sequencing (scRNA-seq) are two important high-throughput sequencing platforms that have wide applications in biomedical research. Bulk RNA-seq reflects the average gene expression of all cells in the sample at a low experimental cost, whereas scRNA-seq enables transcriptomics profiling at a single-cell level, although with higher experimental costs. To integrate the strengths of both sequencing approaches and capitalize on the wealth of existing bulk RNA-seq datasets, we developed a U-Net-based deep learning algorithm, BLUE, to deconvolve bulk RNA-seq samples into cell-type proportions and cell-type-specific gene expression profiles. Built upon a U-Net backbone, BLUE leverages its powerful feature extraction and representation learning capabilities to achieve accurate predictions for cell-type-specific gene expression profiles, which significantly outperform existing deconvolution algorithms. Given the accurate prediction from BLUE, we developed an integrative framework for subtyping cancer patients and identifying cell-type-specific gene signatures that can function as prognostic biomarkers for cancer.

## Author summary

Understanding the behavior of different cell types in the human body is crucial for advancing medical research, especially in areas such as cancer diagnosis and treatment. Scientists use powerful sequencing technologies to study gene expression activity, either by examining all the cells in a sample together (which is affordable but less detailed) or by analyzing individual cells (which offers more detailed information but is significantly more expensive). In our work, we aim to combine the advantages of both worlds.

We developed a novel computational algorithm, BLUE, to “unmix” cell-type-level information from cheaper, bulk samples and reveal what is happening inside each cell type. We adapted U-Net to uncover cell-specific gene activity with high accuracy.

Using BLUE, we can now make better use of the large amounts of existing bulk data to study diseases like cancer. For example, we can identify different groups of patients based on their cell activity and discover specific gene patterns that may help predict how a person’s cancer will progress. This can facilitate better understanding of cancer biology and ultimately support more personalized and effective treatment strategies.

## 1 Introduction

Bulk RNA sequencing has been widely used in clinical applications such as cancer classification, biomarker identification, disease diagnosis, and prognosis analysis [1]. Since RNA molecules from all cells in one bulk sample were mixed together before sequencing, bulk RNA-seq data reflects an average gene expression profile for a collection of cells in one bulk sample [2], which normally includes multiple cell types with varying gene expression profiles. On the other hand, single-cell sequencing (scRNA-seq) data provides gene expression profiles for thousands or more individual cells in one sample [3], revealing the heterogeneity of gene expressions at a single-cell resolution. It is particularly beneficial for the transcriptomic characterization of tumor samples, which typically exhibit exceptional transcriptional diversity [1]. However, due to higher experimental costs, scRNA-seq datasets are less prevalent and less accessible compared to bulk RNA-seq datasets. In addition, scRNA-seq studies typically have small sample sizes and exhibit high variability within individual samples and across different samples, making them statistically less reliable for identifying cancer patient subtypes or prognostic biomarkers.

To combine the large sample size in easily accessible bulk RNA-seq and high-resolution gene profiling in scRNA-seq, researchers have developed bulk deconvolution algorithms to decompose bulk samples into specific cell-type compositions. Quantification of cell-type proportions in bulk samples serves as an important approach to understand intra-tumor heterogeneity, tumor microenvironment [2], and identification of therapeutical targets [4]. In the existing literature, bulk deconvolution methods typically model one bulk sample as a linear mixture of various cell types, with cell-type proportions being the mixing weight that could be solved by conventional optimization algorithms such as linear support vector regression in CIBERSORTx [5,6], matrix decomposition in SCDC [7] and BayesPrism [8], weighted non-negative least squares regression in DWLS [9] MuSiC [10] and Bisque [11], or machine learning models such as fully connected neural networks in Scaden [12] and deep autoencoder in TAPE [13]. While existing algorithms can achieve decent accuracy when predicting cell-type proportions in bulk RNA-seq datasets, most existing algorithms either do not achieve accurate predictions of cell-type-specific gene expression profiles (GEPs) in bulk samples, or were not designed to make such predictions. As a result, the utility of predicted cell-type-specific GEPs in facilitating new discoveries in downstream tasks is underexplored in the literature.

To address the challenges mentioned above, we proposed BLUE, **B**ulk RNA-seq deconvo**L**ution via a **U**-Net based d**E**ep learning model, for accurate predictions of cell-type proportion and cell-type-specific GEPs. BLUE is a novel deep learning model specifically tailored for disentangling the predictions of cell-type proportions and GEPs for every cell type of interest. We demonstrated BLUE’s accuracy and robustness under various conditions on multiple simulated or real-world bulk RNA-seq datasets. Based on the predicted cell-type-specific GEPs from BLUE, we developed an integrative framework for patient subtyping using cell-type-specific gene signatures. We applied BLUE to deconvolve bulk RNA-seq samples of Acute Myeloid Leukemia (AML) patients in The Cancer Genome Atlas (TCGA). The cell-type-specific gene expression signatures generated by BLUE identified three patient subtypes with distinctive survival outcomes in TCGA, and these patient subtypes were validated in an independent AML cohort in the Therapeutically Applicable Research to Generate Effective Treatments (TARGET) study, demonstrating that BLUE’s predicted cell-type-specific GEPs provided biologically meaningful insights and prognostic biomarkers.

## 2 Results

**Fig 1:**
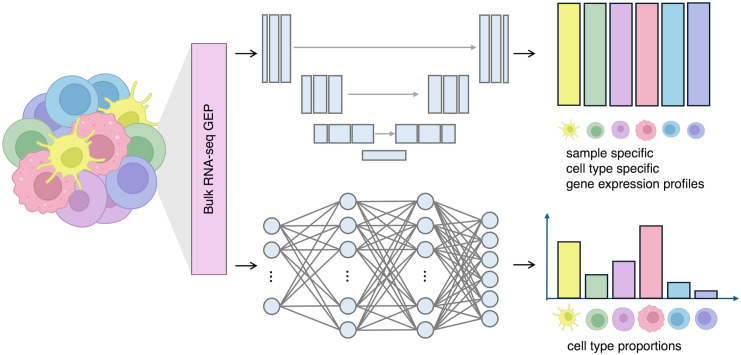
Architecture of BLUE. The input is the gene expression vector of a bulk sample. BLUE contains two branches. The top branch in the figure is a U-Net model to output cell-type-specific gene expression profiles. The bottom branch in the figure is a 5-layer fully connected MLP to output cell-type proportions. The two branches are jointly trained end-to-end on pseudobulk RNA-seq samples simulated from scRNA-seq reference datasets.

The network architecture of BLUE is specifically designed to disentangle the prediction of cell-type proportions and the prediction of gene expression profiles for each cell type. The input to the BLUE model is the gene expression vector of a bulk sample. Although there is no limit for the number of genes in the training process, a subset of the whole transcriptome is normally selected as the input features to make the network training computationally efficient, for example, selecting genes that are differentially expressed among the cell types to be deconvolved. The gene expression profile of the selected genes serves as the input features of BLUE and is then passed into a 5-layer fully connected multilayer perceptrons (MLP) (bottom branch in Fig 1) to obtain cell-type proportions of the input bulk sample. The top branch in Fig 1 is adapted from the U-Net model to deconvolve the input gene expression profile into cell-type-specific gene expression profiles.

The model is trained in a supervised manner on simulated bulk RNA-seq data, which we termed as pseudobulk RNA-seq data. We used multiple scRNA-seq reference datasets to simulate training and validation data. These scRNA-seq reference datasets were generated from the same tissue source as the bulk RNA-seq data and contained cell-type annotations. All cells in one scRNA-seq reference were randomly split into training, validation and test groups to avoid potential information leakage. Cell-type proportions were simulated via Dirichlet distributions with different choices of parameter α, which ensured the diversity of simulated cell-type proportions to include situations such as imbalanced or rare cell types. For every simulated cell-type proportion, cells were randomly sampled from one split (training or validation or test group) and the average gene expression of all sampled cells was computed to simulate one pseudobulk RNA-seq sample. We also recorded the ground truth cell-type-specific GEPs for every simulated bulk sample by averaging gene expressions from sampled cells of the same cell type. The training pseudobulk samples were library-size normalized (divided by the total number of counts in the sample and then multiplied by a scaling factor 10^6^) and then $\log_{2}(\cdot +1)$ transformed. Without other normalization steps that might lead to potential information loss, the predicted GEPs from BLUE are in the same log2 space as the training data, which could be easily transformed into the library-size normalized count space if needed. The training loss is a weighted sum of two Mean Squared Error (MSE) losses, one for the reconstruction error between predicted and ground truth cell-type-specific gene expression profiles, and the other for the prediction error between ground truth and predicted cell-type proportions. After training, BLUE is able to deconvolve any bulk RNA-seq sample into cell-type proportions for the cell types involved in the training process and their corresponding cell-type-specific GEPs.

### 2.1 Evaluating BLUE on PBMC datasets

**Fig 2:**
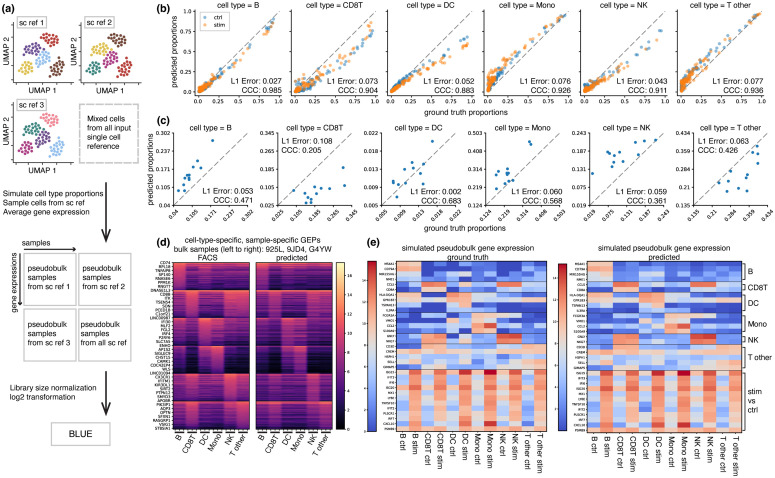
BLUE achieves high correlations with ground truth in simulated and real-world datasets of PBMC. **a,** The workflow for simulating bulk samples from scRNA-seq reference datasets. Training and validation pseudobulk samples were simulated from three publicly available PBMC scRAN-seq datasets. **b,** Scatter plot of predicted cell-type proportions vs. ground truth proportions for randomly generated 100 PBMC ctrl pseudobulk samples (blue dots) and 100 PBMC stim pseudobulk samples (orange dots). *L*_1_ error and CCC score are shown in each subfigure. **c,** Scatter plot of predicted cell-type proportions vs. ground truth proportions for real PBMC bulk samples with experimentally measured cell-type proportions serving as the ground truth. **d,** Heatmap (in $\log_{2}$ scale) of predicted cell-type-specific gene expression profiles for three real bulk samples: 925L, 9JD4, G4YW. The left heatmap is cell-type-specific GEPs measured by bulk RNA-seq of FACS-sorted cell types. The right heatmap is the cell-type-specific GEPs predicted by BLUE. Each column is one GEP vector for one cell type in one sample. Rows are cell-type-specific DEGs ordered by their expression level within the cell type. The top 300 DEGs for each cell type are visualized. **e,** Heatmap (in $\log_{2}$ scale) of predicted cell-type-specific GEPs for the average of simulated bulk samples. Each column is the average of gene expressions of the same cell type from all 100 simulated samples under the same condition. Rows are genes differentially expressed among different cell types or between two conditions (ctrl vs. stim).

We applied BLUE in the context of Peripheral Blood Mononuclear Cells (PBMC) to demonstrate BLUE’s ability to accurately predict cell-type proportions and cell-type-specific GEPs. After training BLUE using pseudobulk samples simulated from three publicly available PBMC scRNA-seq datasets, we evaluated BLUE on both simulated PBMC pseudobulk samples and real PBMC bulk RNA-seq samples from multiple independent studies. The three PBMC scRNA-seq reference datasets, PBMC 6k/8k/10k, were downloaded from 10x Genomics public database. These scRNA-seq reference datasets were annotated into six cell types: B cells (B), CD8T cells (CD8T), other T cells (T other), Dendritic Cells (DC), Monocytes (Mono), Natural Killer (NK). Training and validation pseudobulk RNA-seq samples were constructed by cells from each scRNA-seq reference dataset. To alleviate the batch effect among different scRNA-seq reference datasets, we also simulated training pseudobulk samples by mixing training cells from the three scRNA-seq datasets (Fig 2a). Differentially expressed genes (DEGs) across different cell types were selected as input training features.

We tested BLUE on pseudobulk samples simulated by scRNA-seq datasets from another study [14], which provides two PBMC scRNA-seq datasets under two different conditions: unperturbed PBMC healthy condition (PBMC ctrl) and interferon-stimulated condition (PBMC stim). Half of the test pseudobulk samples were simulated using healthy, unperturbed PBMC cells and the other half were simulated using interferon-stimulated cells. Fig 2b shows the scatter plots of predicted cell-type proportions against the simulated ground truth cell-type proportions for 200 test pseudobulk samples, with blue dots representing PBMC ctrl samples and orange dots as PBMC stim samples. BLUE achieves high Lin’s Concordance Correlation Coefficient (CCC) [15] and low *L*_1_ error, regardless of different sample conditions. This result illustrates that though we trained BLUE on only normal PBMC cells from independent studies, the model generalized well to different biological conditions with distinct gene expression patterns, which demonstrated BLUE’s robustness to different sample conditions, as well as technical variations and batch effect across different studies.

We also applied BLUE to one real bulk RNA-seq dataset, after the model was trained on PBMC 6k/8k/10k reference datasets. The real bulk PMBC dataset [16] contained bulk RNA-seq samples for 12 healthy people, with ground truth cell-type proportions obtained experimentally via Fluorescence-Activated Cell Sorting (FACS). Fig 2c shows the comparison between predicted and ground truth cell-type proportions for the 12 real bulk samples. We observed that BLUE maintained high CCC and low *L*_1_ error in predicting cell-type proportions for real bulk RNA-seq samples.

Quantitative comparisons between BLUE and other existing deconvolution methods are shown in Fig A, Table A, and Table B in S1 Text.

In addition to accurate predictions of cell-type proportions, BLUE accurately predicts meaningful cell-type-specific GEPs in real-world settings. The left subfigure in Fig 2d shows real cell-type-specific GEPs for three bulk samples (925L, 9JD4, G4YW) experimentally obtained by bulk RNA-seq on FACS sorted cell types for each sample. We observed that the predicted sample-specific cell-type-specific GEPs (right subfigure in Fig 2d) matched the expression patterns in the experimentally measured cell-type-specific GEPs in those samples. Quantitative evaluation metrics are listed in Table C and Table D in S1 Text.

BLUE accurately captured distinct gene expression patterns between control and stimulated conditions in the simulation settings. We trained and tested BLUE on pseudobulk samples under two conditions, which were simulated from PBMC ctrl and PBMC stim cells, respectively. Fig 2e shows the ground truth and predicted gene expression profiles averaged across samples under the same condition. The prediction accurately recovers DEGs across different cell types and DEGs between control and stimulated conditions, demonstrating BLUE’s ability to automatically capture gene expression variations based on input bulk RNA-seq profiles only.

### 2.2 Evaluating BLUE on pancreatic islet datasets

To further demonstrate that BLUE accurately captures distinct gene expression patterns in bulk RNA-seq samples under different biological conditions, we applied BLUE to a pancreatic islet dataset [17] that contains scRNA-seq data profiled by the Smart-seq2 for six healthy subjects (H1-H6) and four Type 2 diabetes patients (T2D1 - T2D4), and bulk RNA-seq profiles for three of the six healthy subjects (H3, H4, H6) and all four T2D patients (T2D1 - T2D4). Training and validation pseudobulk samples were simulated using five scRNA-seq datasets, including three healthy subjects and two T2D patients (H1, H2, H5, T2D3, T2D4). After training, BLUE was applied to deconvolve real bulk RNA-seq data from the remaining subjects (H3, H4, H6, T2D1, T2D2). We treated the scRNA-seq profiling of H3, H4, H6, T2D1, T2D2, as the ground truth cell-type-specific GEPs. We selected six most abundant cell types for deconvolution: alpha, beta, gamma, delta, acinar, ductal cells. More details about training are in Methods.

**Fig 3:**
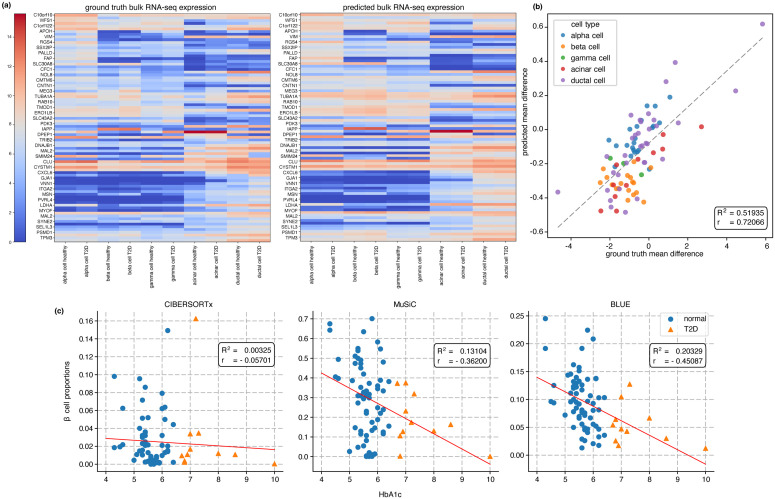
BLUE captures distinct gene expression patterns in real bulk RNA-seq datasets and outperforms existing deconvolution methods. **a,** Gene expression values of healthy and T2D conditions (in $\log_{2}$ space). Each column is a cell-type-specific GEP averaged over cells of the same cell type in all samples under one condition (healthy or T2D). Each row is a gene that Segerstolpe *et al.* selected to be differentially expressed between two conditions. **b,** Scatter plot of the gene expression difference. The x-axis is ground truth differences calculated by subtracting the average expression profile under T2D condition from the average expression profile under the healthy condition. The y-axis is the predicted differences calculated in the same way as x-axis. The dashed line is the linear fitting between y and x, with a linear fitting score *R*^2^ = 0.52 and pearson correlation *r* = 0.72. **c,** Scatter plot of predicted proportions for beta cell vs. hemoglobin A1c (HbA1c) level. From the left subfigure to the right are predictions from CIBERSORTx, MuSiC, BLUE. Red lines are linear regression results. Linear fitting score *R*^2^ and pearson correlation *r* are shown in each plot. Blue dots are normal HbA1c level (under 6.5) while yellow triangles are HbA1c level that corresponds to T2D condition.

Fig 3a shows the average cell-type-specific gene expression levels for healthy subjects and T2D patients as columns. DEGs between healthy and T2D conditions for each cell type were selected by Segerstolpe *et al.* and shown in the heatmap Fig 3a as rows. The predicted gene expressions (right heatmap) show clear patterns between different cell types that match the expression patterns in the ground truth data (left heatmap), which indicates that BLUE’s prediction of cell-type-specific GEP correctly captured the expression variations among different cell types. Since the expression variation within each cell type between healthy and T2D conditions was much smaller than the variation among different cell types, neither the ground truth nor the predicted cell-type-specific GEPs in Fig 3a clearly visualized the differences between the two conditions. Therefore, to better visualize the expression difference between healthy and T2D conditions, Fig 3b showed the predicted gene expression difference against the expression difference in the ground truth data. For each cell type, the difference is calculated by subtracting the average expression profile under T2D condition from the average expression profile under the healthy condition (difference between two adjacent columns that correspond to the same cell type in heatmaps in Fig 3a). In Fig 3b the predicted mean difference achieves a high correlation with the ground truth mean difference (with pearson correlation *r* = 0.72066). This correlation demonstrated that BLUE’s prediction of cell-type-specific GEP accurately captured the cell-type-specific expression variations specific to different biological conditions. More benchmarking metrics for each sample-specific GEPs are included in Table E and Table F in S1 Text.

To compare BLUE with existing deconvolution algorithms in terms of predicting cell-type proportions for real bulk samples, we applied BLUE to deconvolve bulk RNA-seq samples of human pancreatic islets from 89 donors with and without Type 2 diabetes in Fadista *et al.*’s study [18] after training BLUE on the pancreatic islet dataset described above. We compared the predicted cell-type proportions from CIBERSORTx [6] (see Fig B in S1 Text), MuSiC [10] (see Fig C in S1 Text) and BLUE. Since it is known in the literature that hemoglobin A1c (HbA1c) level is an important biomarker for Type 2 diabetes (T2D), cell proportion for beta cells is expected to correlate with HbA1c level negatively [10]. As is shown in Fig 3c, the prediction from BLUE for beta cell-type proportions reflects the expected negative correlation with HbA1c level, with a higher *R*^2^ in linear fitting score and better pearson correlation than MuSiC, while CIBERSORTx failed to recover the expected negative relationship. This further illustrated the prediction of cell-type proportions from BLUE aligns with the underlying biology, and BLUE outperformed existing deconvolution methods such as CIBERSORTx and MuSiC.

### 2.3 Applying BLUE to Acute Myeloid Leukemia for patient subtyping and discovering cell-type-specific gene expression biomarkers

To demonstrate how the predicted cell-type-specific GEPs from BLUE unlock the potential of existing bulk RNA-seq datasets in discovering cell-type-specific prognostic biomarkers, we applied BLUE to the context of Acute Myeloid Leukemia (AML), which is a heterogeneous disease with various clinical presentations and non-uniform responses to treatment. A previous AML study [19] provided scRNA-seq data from 12 AML patients and 4 healthy donors, as well as cell-type annotations of 6 malignant cell types and 9 normal cell types [19]. The 6 malignant cell types were named based on their similarity to normal cell types: hematopoietic stem cell (HSC)-like cells, progenitor (Prog)-like cells, granulocyte-macrophage progenitor (GMP)-like cells, promonocyte (ProMono)-like cells, monocyte (Mono)-like cells, conventional dendritic cell (cDC)-like cells.

**Fig 4:**
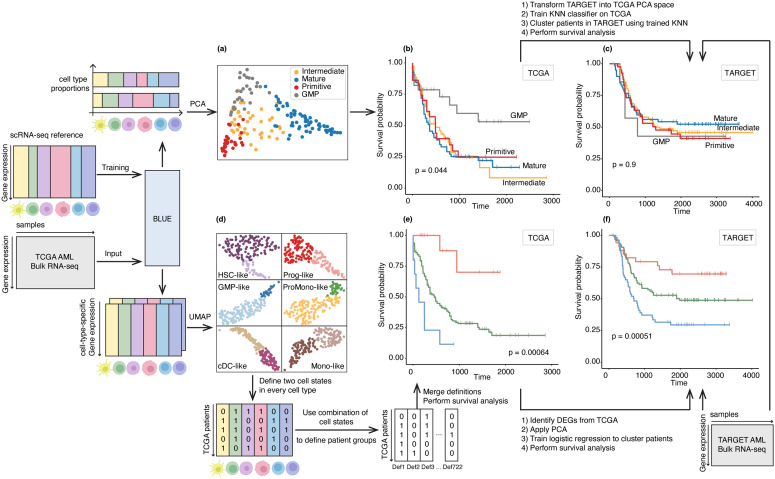
An integrative framework for patient subtyping based on the output from BLUE. BLUE was trained on one independent AML scRNA-seq reference dataset from [19] and applied to deconvolve TCGA-AML dataset into cell-type proportions and sample-specific, cell-type-specific GEPs. The top branch defined patient groups based on predicted cell-type proportions, similar to the analysis workflow in one previous study [22]. GMP patient group exhibits the most favorable prognosis outcome compared to the other three patient groups in the TCGA-AML cohort. The survival pattern failed to be validated in the TARGET-AML cohort. The bottom branch defines TCGA patient groups based on the predicted cell-type-specific GEPs and the resulting three groups exhibited distinct survival differences. The same survival pattern was also observed in TARGET-AML patient cohort, after applying a simple classification pipeline on selected gene expressions to classify TARGET-AML patients.

We applied BLUE to deconvolve AML bulk samples in The Cancer Genome Atlas (TCGA-AML) [20]. Given the predicted cell-type-specific GEPs from BLUE, PCA was applied to transform the predicted cell-type proportions and Leiden Clustering was applied to the PCA space to group patients into 4 clusters (Primitive, GMP, Intermediate, Mature) (Fig 4a). We observed that one patient group with GMP-like malignant cells being the dominant cell type exhibited a distinctively favorable survival outcome compared to the other patient groups (Fig 4b). To validate this finding in an independent cohort, we applied BLUE to predict cell-type proportions for AML patients in the Therapeutically Applicable Research to Generate Effective Treatments (TARGET) cohort [21]. The predicted cell-type proportions for the TARGET-AML cohort were transformed into the PCA space in the TCGA-AML analysis. A k-nearest neighbor (KNN) classifier was trained in the PCA space of the TCGA-AML cohort and applied to the PCA-transformed predicted cell-type proportions for TARGET-AML cohort to classify TARGET-AML patients into the four patient groups. The survival outcomes of the resulting patient groups in TARGET-AML cohort were plotted in Fig 4c. The GMP patient group no longer showed distinguishable survival outcome from the rest of the patient groups. In the TCGA-AML cohort, one of the four patient groups defined by predicted cell-type proportions displayed a more favorable survival outcome than the other groups, which implied that cell-type proportions might be a survival biomarker. However, such a survival difference was not validated on the TARGET-AML cohort, demonstrating that predicted cell-type composition was not a generalizable survival biomarker. In a previous work [22], a similar patient clustering analysis was performed based on the predicted cell-type proportions from CIBERSORTx, which were highly correlated with BLUE’s prediction for cell-type proportion (Fig D in S1 Text). A survival pattern that is similar to Fig 4b was also observed for TCGA-AML cohort and failed to generalize to TARGET-AML cohort either. This further confirmed our observation that cell-type proportion is not a robust, generalizable survival biomarker in the context of AML.

In order to obtain a more robust and generalizable survival biomarker for AML, we designed a framework to cluster AML patients based on cell-type-specific GEPs (see the bottom branch in Fig 4). Based on the predicted cell-type-specific GEPs for each cell type, patients in the TCGA-AML cohort were stratified into two groups that reflect two cell states of the cell type, as shown in the UMAPs in Fig 4d. Similar to how protein marker combinations are used to define various cell types in immunology, we used combinations of cell states of the 6 malignant cell types to define patient groups (Fig 4d → e). Among all $\sum_{i=1}^{6}2^{i}\;C_{i}^{6}$ possible ways to define patient groups based on binary cell states from every malignant cell type, we applied multiple hypothesis testing and identified 37 unique patient group definitions with significant survival differences (Benjamini–Hochberg adjusted p-value < 0.05). These 37 definitions were further reduced by merging similar definitions via pairwise intersection over union (IoU) metric and consensus patients among similar definitions (see details in Methods). Finally, we identified two patient groups, one patient group with the most favorable prognosis outcomes and one patient group with the most unfavorable prognosis outcomes (p-value: 0.00064, see Fig 4e).

Then we validated the most favorable and unfavorable prognosis groups using the TARGET-AML cohort. We identified DEGs between the most favorable prognosis TCGA-AML patient group and the rest TCGA-AML patients and applied PCA transformation to the selected bulk gene expression data of the DEGs. A logistic regression classifier was trained on PCA-transformed TCGA-AML gene expressions and directly applied to PCA-transformed TARGET-AML gene expressions to identify the most favorable prognosis group (details see Methods). The same procedure was repeated for the most unfavorable prognosis TCGA-AML patient group. The resulting most favorable and unfavorable prognosis groups in the TARGET-AML cohort shared the same survival pattern as the TCGA dataset with a significant p-value of 0.00051 (Fig 4f).

We examined known clinical prognostic markers in our identified TCGA AML patient groups with distinct survival. The most favorable prognosis patient group is enriched by patients with low cytogenetic risk compared to the other two patient groups, while the most unfavorable prognosis patient group is composed of patients with intermediate and high cytogenetic risk. We also examined French-American-British (FAB) classification and FLT3 Mutation, and did not observe any correlation between our identified patient groups with these two clinical markers. Therefore, our classification of TCGA patients is partially concordant with but could not be fully explained by existing clinical markers. The Cox model hazard ratio analysis in Fig E in S1 Text also demonstrates the uniqueness of the new patient subtyping and its power to reduce or increase hazard compared with other existing clinical markers. All the analyses above further indicate that our patient subtyping based on cell-type-specific GEPs contributes novel viewpoints to the current AML patient classification system.

We further demonstrated the value of cell-type-specific gene expression in identifying predictive prognostic biomarkers. Differentially expressed genes from cell-type-specific GEPs predicted by BLUE were analyzed for Gene Ontology (GO) terms using PANTHER & Enrichr (Reactome version 86). From the gene expression profiles of GMP-like cells, 2715 reciprocally regulated genes were identified, which were up-regulated in the most favorable prognosis patient group and simultaneously down-regulated in the most unfavorable prognosis patient group. The pathway analysis on those 2715 reciprocally regulated genes highlighted increases across the entire ubiquitin-proteasome system (UPS; WikiPathway 2023 Human, Proteasome degradation WP183, $p=2.7\times 10^{-11}$), which included both autophagy and deubiquitinases (DUBs). Specific proteasome targets were also identified, such as tumor suppressors (p53 and Pten). Therefore, increased priming toward cell differentiation and death is more active in GMP-like malignant cells in the most favorable prognosis patient group.

Interestingly, from the GSEA analysis of the DEGs from BLUE’s predicted specific gene expression profiles, Immune System R-HAS-168256 (Reactome 2022) was highly upregulated ($p=2.7\times 10^{-48}$). Therefore, it is plausible that the immunoproteasome may be particularly relevant because of the abundance of pro-inflammatory gene sets that are also co-regulated with the UPS in the most favorable prognosis patients. This discovery also resonates with one previous study that correlated patients’ improved survival outcomes in NSCLC and melanoma with overexpressed immunoproteasomes [23]. Importantly, the UPS has almost exclusively been targeted for inhibition by current therapies, and surprisingly, our analysis suggests that increased UPS activity in best-responding patients could be biologically favorable. Therefore, this powerful cell-type-specific GEP analysis reveals that proteasome activation should be induced rather than inhibited to achieve better survival outcomes. This remains a subject for our future investigation. The utility of cell-type-specific GEPs predicted from BLUE has already been illustrated since the downstream analysis based on those predicted profiles provides us with potentially meaningful biological insights into the complex immune microenvironment of AML.

## 3 Discussion

BLUE is a deep-learning-based deconvolution algorithm that accurately predicts cell-type proportions and cell-type-specific gene expression profiles for bulk gene expression samples. As shown in various simulated and real-world scenarios, BLUE is robust in dealing with datasets from different sequencing platforms and under various biological conditions in real-world settings. The predicted cell-type-specific GEPs uncover the cellular heterogeneity in tumor samples, which represents a new type of cancer biomarker. BLUE’s accurate prediction of cell-type-specific GEPs paves the way for the development of a universal, integrative framework for identifying and validating cell-type-specific gene expression signatures that can serve as predictive and prognostic cancer biomarkers.

As we have demonstrated in the context of AML, GSEA analysis of BLUE’s predicted cell-type-specific GEPs and DEGs detected well-known pathways and highlighted the importance of immunoproteasome for future investigation. Additionally, proteasome activity in bulk AML cells, as analyzed by UALCAN [24] on a gene-by-gene basis, shows worse survival outcomes for individual UPS genes. However, predicted GMP-like and HSC-like AML GEPs and DEGs share unique patient survival outcomes from those of the bulk. It has been reported in previous literature that cancer stem cells (CSCs) have lower proteasome activity compared to bulk tumors [25–28]. So, these GEP differences may reflect the unique biology of CSCs vs. bulk cancer [29]. This illustrates that BLUE creates opportunities for more in-depth examinations of existing abundant bulk RNA-seq datasets at a resolution of the cell-type level. The predicted cell-type-specific GEPs from BLUE facilitate the discovery of cell-type-related gene signatures and consequently enable a more thorough investigation of tumor heterogeneity and cell-type-specific changes associated with the disease.

## 4 Conclusions

Throughout this work, we demonstrate BLUE’s ability to generate biologically meaningful, cell-type-specific GEPs for real bulk RNA-seq samples, provided that the samples share the same biological context as the scRNA-seq datasets used for training. Compared with existing deconvolution methods that also infer cell-type-specific GEPs, BLUE achieves higher fidelity to ground-truth profiles and produces outputs directly in the expression space without distortion from irreversible transformations. Moreover, BLUE supports a variety of sequencing platforms (Table G in S1 Text) and flexible customization of both input and output gene lists, enabling broader applications in the downstream tasks.

We also acknowledge several limitations of the current framework. To fully realize BLUE’s potential, access to high-quality scRNA-seq datasets from matched biological contexts (i.e., health condition, tissue type, and predefined cell types of interest) is essential. In addition, BLUE is currently trained in a context-specific manner, requiring retraining for each new biological setting. One direction for future work is to extend BLUE into a foundation model that can generalize across diverse biological contexts without requiring task-specific retraining.

In summary, we developed a novel deep-learning-based algorithm, BLUE, for bulk deconvolution. By accurately predicting the cell-type proportions and cell-type-specific gene expression profiles, BLUE opens up new possibilities for re-analyzing existing abundant bulk RNA-seq data at a more granular, cell-type level, while inheriting the advantage of a large sample size and enriched clinical information related to bulk RNA-seq samples. Consequently, BLUE establishes a foundation for developing an integrative framework for gaining novel biologically meaningful insights with prognostic and therapeutic implications from transcriptomics data.

## 5 Methods

### 5.1 Modeling bulk RNA-seq deconvolution

For a bulk RNA-seq sample $𝐛\in {\mathbb{R}}^{g\times 1}$ of *g* genes composed of *c* cell types, let $𝐩\in {\mathbb{R}}^{c\times 1}$ denote the cell-type proportions in the bulk sample **b**, and $𝐆\in {\mathbb{R}}^{g\times c}$ denote the underlyin*g* cell-type-specific GEPs for *c* cell types in **b**. Under the assumption of linearity, we have


𝐆𝐩=𝐛

BLUE predicts both the cell-type proportion $𝐩\in {\mathbb{R}}^{c\times 1}$ and cell-type-specific GEPs $𝐆\in {\mathbb{R}}^{g\times c}$ for any input bulk sample $𝐛\in {\mathbb{R}}^{g\times 1}$.

### 5.2 Simulation and preprocessing of bulk samples

All the input bulk samples, real or simulated, are library-size normalized (divided by the number of all counts in one sample) and then multiplied by a size factor of 10^6^. The normalized counts are then transformed by log(·+1) operation. No other transformation is applied.

The total number of pseudobulk samples simulated for training BLUE is dependent on the number of cell types involved in deconvolution, and the number of total cells in scRNA-seq datasets. The training pseudobulk samples cover various cell-type proportions simulated via Dirichlet distributions with different choices of parameter α, which ensures the diversity of simulated cell-type proportions to include situations such as imbalanced or rare cell types. For more experiment details regarding parameter settings, see following sections.

### 5.3 Feature selection

In our experiments, we normally selected differentially expressed genes across cell types as the input features, as well as genes of interest such as DEGs across different sample conditions. We operated BLUE on the same set of genes for input and output GEPs, and the maximum number of output genes that we tested BLUE on is 9k. For more experiment details regarding parameter settings, see following sections.

### 5.4 Model architecture and training setup

The input bulk gene vectors are passed through one linear layer of dimension 4096 for feature extraction. To predict cell-type proportions, the extracted features are passed through a 5-layer MLP with size 4096, 1024, 256, 64, and the number of cell types. The activation function between linear layers is SiLU. For the U-Net branch, the extracted features are passed into 4 downsampling blocks and 4 upsampling blocks. Each downsampling block consists of one 1D max pooling layer, and two rounds of application of 1D convolution, 1D batch normalization and ReLU activation function. Each upsampling block consists of one 1D transposed convolution operator, and two rounds of application of 1D convolution, 1D batch normalization and ReLU activation function. One downsampling block doubles the channels and halves the feature dimensions while the upsampling block does the opposite. The output of U-Net is cell-type-specific GEPs with size number of cell types by number of predicted genes, and the channel dimension in the output of the final upsampling block corresponds to individual cell types.

The training optimizer is Adam. The loss function is the weighted summation of the MSE loss between the predicted and ground truth cell-type proportions and the MSE loss between the predicted and ground truth cell-type-specific GEPs. The weight between two MSE losses is applied to balance loss values for joint end-to-end training of two branches.

### 5.5 Metrics

The *L*_1_ error between ground truth *x* and prediction *y* is calculated by


$$\frac{1}{N}\sum_{i=1}^{N}|y_{i}-x_{i}|$$

Lin’s Concordance Correlation Coefficient (CCC) is calculated by


$$\rho_{c}=\frac{2\rho \sigma_{x}\sigma_{y}}{\sigma_{x}^{2}+\sigma_{y}^{2}+(\mu_{x}-\mu_{y}{)}^{2}}$$

where $\mu_{x}$ and $\mu_{y}$ are the means and $\sigma_{x}^{2}$ and $\sigma_{y}^{2}$ are the corresponding variances for ground truth *x* and prediction *y*. $\rho =\rho_{x,y}=\frac{𝔼[(x-\mu_{x})(y-\mu_{y})]}{\sigma_{x}\sigma_{y}}$ is the pearson’s correlation coefficient.

The linear fitting score in Fig 3 is calculated by


$$R^{2}=1-\frac{\sum_{i}^{N}(y_{i}-x_{i}{)}^{2}}{\sum_{i}^{N}(x_{i}-\overset{―}{x})}$$

where $\overset{―}{x}$ is the mean of ground truth *x*. The best possible score is 1 and a constant prediction would get a *R*^2^ score of 0.

### 5.6 PBMC experiment details

In the experiment that generates results in Fig 2b, 2c, 2d, BLUE was trained on three normal PBMC reference datasets: PBMC 6k/8k/10k. All scRNA-seq datasets were annotated into six cell types following the standard pipeline in python package *Scanpy*: B cells (B), CD8T cells (CD8T), other T cells (T other), Dendritic Cells (DC), Monocytes (Mono), Natural Killer (NK). Cells were randomly split into training, validation and test groups (ratio: 8:1:1) to avoid information leakage during pseudobulk simulation. Cell-type proportions were simulated for training, validation and test pseudobulk samples using Dirichlet distribution with α=25 (evenly distributed proportions) and 7 (one dominant cell type). Then, cells from the same scRNA-seq dataset were randomly sampled according to every randomly generated cell-type proportion, and their average expression was computed to simulate one pseudobulk sample. Besides pseudobulk samples simulated from single scRNA-seq dataset, roughly a quarter of the total training pseudobulk samples were simulated by sampling from all training cells in all three scRNA-seq datasets. In total, 20,400 training pseudobulk samples were simulated.

For every scRNA-seq reference dataset, cell-type-specific DEGs were selected using Wilcoxon rank-sum test to compare one cell type versus all the other cell types in the same sample. After applying a threshold to the Benjamini-Hochberg adjusted p-value (p-value < 0.1) and requiring a positive log fold change, we sorted the remaining genes by their log fold change in descending order and selected the top 285 genes for every cell-type-specific DEG list. The union of the DEG lists from each cell type in each scRNA-seq reference dataset formed the final gene list used for the training and prediction, resulting in a total of 3000 genes. In our experiments, the prediction results from BLUE were independent of the number of genes involved in the training of BLUE. Our results presented above used 3k genes in the training and inference.

In the experiments for Fig 2e, cells in every scRNA-seq dataset were first split into training, validation and test groups (ratio = 8:1:1) before simulating pseudobulk samples. The model was trained on pseudobulk samples simulated using training cells from five PBMC scRNA-seq datasets: PBMC 6k/8k/10k/ctrl/stim, and the evaluations were performed on pseudobulk samples simulated using test cells from PBMC ctrl/stim datasets. Other training procedures were the same as those in the previous PBMC experiment.

### 5.7 Pancreatic islets experiment details

In the experiment of deconvolving 89 external bulk RNA-seq datasets, all six healthy samples and four T2D patients were used in the training process (Fig 2c). Since the number of cells was at the level of hundreds for each scRNA-seq sample, we decided to simulate pseudobulk samples for each health condition rather than independently simulate pseudobulk samples for each individual donor. Cells from all three healthy people were merged into one batch and cells from two T2D patients were treated as another batch. Thus we had in total two scRNA-seq references to simulate pseudobulk samples.

In the paper [17] that published this dataset, differential expression analysis between cell types (α, β, γ, δ, acinar, and ductal cells) was performed using one-way ANOVA with log2-transformed expression data from the five healthy male donors. DEGs were provided as a supplementary table and were used in our training and prediction.

In the experiment to test the generated cell-type-specific GEPs (Fig 2a, 2b), three healthy and two T2D patient scRNA-seq datasets (H3, H4, H6, T2D1, T2D2) were completely held out for simulating test pseudobulk samples. Training was done on the pseudobulk samples simulated from the remaining scRNA-seq datasets (H1, H2, H5, T2D3, T2D4) and other training details were the same as PBMC experiments.

### 5.8 AML experiment details

The scRNA-seq dataset was annotated into 6 malignant cell types and 9 normal cell types in the previous study [19] and all 6 malignant cell types were used in the deconvolution of TCGA AML bulk RNA-seq dataset: hematopoietic stem cell (HSC)-like cells, progenitor (Prog)-like cells, granulocyte-macrophage progenitor (GMP)-like cells, promonocyte (ProMono)-like cells, monocyte (Mono)-like cells, conventional dendritic cell (cDC)-like cells. All cells from different scRNA-seq samples were mixed together before being split into training, validation and test groups for pseudobulk sample simulation due to the small amount of annotated cells in each scRNA-seq dataset.

All common genes (~9k) between the input single cell reference and TCGA AML bulk were selected as training features and input into BLUE.

For every scRNA-seq reference dataset, cell-type-specific DEGs were selected using Wilcoxon rank-sum test to compare one cell type versus all the other cell types in the same sample. We applied a threshold to the Benjamini-Hochberg adjusted p-value (p-value < 0.1) and required a positive log fold change. The union of the DEG lists from each cell type in each scRNA-seq reference dataset were the final gene list used in the training and prediction, resulting in a total of 3538 genes.

To define patient groups based on predicted cell-type-specific GEPs, for every cell type, Leiden clustering algorithm was applied to the predicted cell-type-specific GEPs under default hyperparameters and resulted in two patient groups in one cell type, which reflected two cell states of one cell type. Similar to how protein marker combinations are used to define various cell types in immunology, we used combinations of cell states of the 6 malignant cell types to define patient groups. Given *k* cell types (k∈1,2,3,4,5,6) with 2 cell states in each cell type, there were 2^k^ ways to define patients. In every definition, *k* cell states were picked from *k* cell types that jointly define one patient group. The rest of the patients automatically form another group. Therefore, there were always two patient groups in every definition formed by any combination of *k* cell types. In total, there were


$$\sum_{i=1}^{6}2^{i}\;C_{i}^{6}=722$$

ways to define patient groups based on predicted cell-type-specific GEPs. 37 unique definitions with distinct survival differences (Benjamini–Hochberg adjusted p-value < 0.05) were candidates for potentially meaningful patient group definitions.

To further reduce the number of definitions, pairwise Intersection over Union (IoU) is calculated for those 37 definitions.

Let | *X* | denote the cardinality of a set *X*. Let *A* and *B* be two patient group definitions and *A*_1_, *A*_2_ be two patient groups in definition $A,\;(|A_{1}|<|A_{2}|)$, *B*_1_, *B*_2_ be two patient groups in definition $B,\;(|B_{1}|<|B_{2}|)$. The similarity between definitions *A* and *B* is defined as the IoU score between *A* and *B*, which is defined as:


$$\text{Similarity score}=\max\left(\frac{\left|A_{1}⋂B_{1}\right|}{\left|A_{1}⋃B_{1}\right|},\frac{\left|A_{1}⋂B_{2}\right|}{\left|A_{1}⋃B_{2}\right|}\right)$$

The similarity score was calculated for each pair of definitions and filled into a symmetric matrix *S*. *S* was then binarized according to the threshold:


$$\begin{array}{c} S_{ii}=\left\{ \begin{array}{ccc} 1 & \text{if} & s_{ii}\geq 0.8\\ 0 & \text{else} \end{array}\right. \end{array}$$

After binarization, matrix *S* defined the adjacency matrix for 37 definitions, where edges were drawn if two definitions have IoU greater than 0.8. This adjacency matrix defined a graph with 6 connected components, indicating that 37 definitions could be further merged and reduced by finding consensus within each connected component.

To further reduce the number of definitions, we identified consensus definition of patient groups among all definitions in the same connected component. Algorithm 1 was applied to identify the correct correspondence of two patient groups in every definition. In other words, to figure out patient group 0 in one definition corresponds to which patient group (0 or 1) in another definition. Algorithm 2 identified consensus patient groups after matching patient groups in different definitions through Algorithm 1.

**Algorithm 1. Identify patient group correspondence among definitions**

C← patient group definition with the smallest p-value in survival analysis

$C_{1},C_{2}\leftarrow$ two patient groups defined by *C*

$v_{1}^{\left(C\right)}\in {\mathbb{R}}^{\text{\#TCGA patients}}\leftarrow v_{1k}^{\left(C\right)}=\left\{ \begin{array}{cc} 1 & \text{if patient}\;k\;\text{in}\;C_{1}\\ 0 & \text{if patient}\;k\;\text{in}\;C_{2} \end{array}\right.$ ▷ denote one patient

group assignment

$v_{2}^{\left(C\right)}\in {\mathbb{R}}^{\text{\#TCGA patients}}\leftarrow v_{2k}^{\left(C\right)}=\left\{ \begin{array}{cc} 0 & \text{if patient}\;k\;\text{in}\;C_{1}\\ 1 & \text{if patient}\;k\;\text{in}\;C_{2} \end{array}\right.$ ▷ denote another patient

group assignment

**For** other definitions *D* in this connected component **do**

 Compute distance for every pair of $v_{i}^{\left(C\right)}$ and $v_{j}^{\left(D\right)}$, i,j∈{1,2}: $d(v_{i}^{\left(C\right)},v_{j}^{\left(D\right)})=\sum_{k}((v_{ik}^{\left(C\right)}+v_{jk}^{\left(D\right)})\;\text{mod}\;2)$

 **if** $d(v_{1}^{\left(C\right)},v_{1}^{\left(D\right)})<d(v_{1}^{\left(C\right)},v_{2}^{\left(D\right)})$ **then**

  $v_{1}^{\left(C\right)}\sim v_{1}^{\left(D\right)}$ ▷ Patient group 0 in $v_{1}^{\left(C\right)}$ corresponds to patient group 0 in $v_{1}^{\left(D\right)}$

 **else if** $d(v_{1}^{\left(C\right)},v_{1}^{\left(D\right)})>d(v_{1}^{\left(C\right)},v_{2}^{\left(D\right)})$

  $v_{1}^{\left(C\right)}\sim v_{2}^{\left(D\right)}$ ▷ Patient group 0 in $v_{1}^{\left(C\right)}$ corresponds to patient group 0 in $v_{2}^{\left(D\right)}$

 **end if**

 **if** $d(v_{2}^{\left(C\right)},v_{1}^{\left(D\right)})<d(v_{2}^{\left(C\right)},v_{2}^{\left(D\right)})$ **then**

  $v_{2}^{\left(C\right)}\sim v_{1}^{\left(D\right)}$ ▷ Patient group 0 in $v_{2}^{\left(C\right)}$ corresponds to patient group 0 in $v_{1}^{\left(D\right)}$

 **else if** $d(v_{2}^{\left(C\right)},v_{1}^{\left(D\right)})>d(v_{2}^{\left(C\right)},v_{2}^{\left(D\right)})$

  $v_{2}^{\left(C\right)}\sim v_{2}^{\left(D\right)}$ ▷ Patient group 0 in $v_{2}^{\left(C\right)}$ corresponds to patient group 0 in $v_{2}^{\left(D\right)}$

 **end if**

**end for**

**return** Similar group assignments $\left\{v_{1}^{\left(C\right)},v_{i_{1}}^{\left(D_{1}\right)},v_{i_{2}}^{\left(D_{2}\right)},\cdots ,v_{i_{n}}^{\left(D_{n}\right)}\},\;1emi_{n}\in \{1,2\},\;D_{n}\in \{\text{definitions in one connected component}\right\}$

& Similar group assignments $\left\{v_{2}^{\left(C\right)},v_{j_{1}}^{\left(D_{1}\right)},v_{j_{2}}^{\left(D_{2}\right)},\cdots ,v_{j_{n}}^{\left(D_{n}\right)}\},\;1emj_{n}\in \{1,2\},\;D_{n}\in \{\text{definitions in one connected component}\right\}$

**Algorithm 2. Find consensus patient assignment**

**Input:** Algorithm 1’s output: patient group assignments: $\left\{v_{1}^{\left(C\right)},v_{i_{1}}^{\left(D_{1}\right)},v_{i_{2}}^{\left(D_{2}\right)},\cdots ,v_{i_{n}}^{\left(D_{n}\right)}\right\}$ or $\left\{v_{2}^{\left(C\right)},v_{j_{1}}^{\left(D_{1}\right)},v_{j_{2}}^{\left(D_{2}\right)},\cdots ,v_{j_{n}}^{\left(D_{n}\right)}\right\}$

**for** patient *k* in TCGA patients **do**

 **if** patient *k* is in group 0 in more than (*n* + 1)/2 definitions **then**

  Assign patient *k* to group 0 in consensus

 **else if** patient *k* is in group 1 in more than (*n* + 1)/2 definitions

  Assign patient *k* to group 1 in consensus

 **else**

  Assign patient *k* to group 2 in consensus

 **end if**

**end for**

Finally, one group assignment surpassed the other if fewer patients were assigned to group 2. We arrived at 6 definitions after merging definitions in the same connected component.

Three survival patterns were evident among the remaining 6 definitions. Four of them defined a patient group with a favorable survival outcome compared to the rest of the patients, while two of them defined a patient group with an unfavorable survival outcome compared to the rest of the patients. After applying another round of majority voting to find consensus patient groups within definitions that share the same survival pattern, we finally settled on a new definition of three patient groups for all TCGA patients.

After we finalized the definition of three TCGA-AML patient groups, we used the TARGET-AML dataset to validate our discovery. DEGs were selected by comparing the favorable prognosis TCGA-AML patient group against the rest of the TCGA-AML patients. PCA (number of component = 1) was applied to the selected DEG expressions for TCGA patients and TARGET patients, respectively. To select the most favorable prognosis group in TARGET-AML cohort, a logistic regression classifier was trained on TCGA-AML patients in the PCA space and directly applied to the PCA-transformed TARGET-AML samples and resulted in two patient groups. Patients in one group exhibited a favorable survival outcome. The unfavorable prognosis patient group was identified in the same way. Despite the classification of patients was done in parallel to each other, no TARGET-AML patient was assigned to both the best and the worst prognosis group at the same time. Finally, we obtained a definition of three patient groups for TARGET-AML cohort, which exhibited the same pattern of survival outcomes as TCGA-AML patient groups.

## Supporting information

S1 Text Supplementary information. (PDF)
