# Supplementary material for: Deconvolving cell-type-specific gene expression profiles from bulk RNA-seq samples
Source: PLoS Comput Biol. 2026 Mar 26;22(3):e1014101. doi: 10.1371/journal.pcbi.1014101 (PMC13038110; doi:10.1371/journal.pcbi.1014101)
Supplement: S1 Text — (PDF) [file pcbi.1014101.s001.pdf]

Supplementary Information

Deconvolving cell-type-specific gene expression profiles from bulk RNA-seq samples

Sichen Zhu[1], Zhengqi Wang[2,3], Kevin Bunting[2,3], Peng Qiu[1*]

[1]Department of Biomedical Engineering, Georgia Institute of Technology and Emory University, Atlanta, Georgia, United States of America.
[2]Department of Pediatrics, Division of Hematology and Oncology, Emory University, Atlanta, Georgia, United States of America.
[3]Aflac Cancer and Blood Disorders Center, Children's Healthcare of Atlanta, School of Medicine, Emory University, Atlanta, Georgia, United States of America.

*Corresponding author(s). E-mail(s): peng.qiu@bme.gatech.edu;
Contributing authors: sichenzhu@gatech.edu; zhengqi.wang@emory.edu; kevin.bunting@emory.edu;

Comparing BLUE and existing deconvolution methods

For the cell-type deconvolution task, we compared the performance of BLUE versus existing deconvolution methods on the datasets used in our experiments. The comparisons are present in the format of scatter plots in Fig A. B. C. D.

Quantitative results are listed in Table A. B. C. D. E. F. Different deconvolution methods have different preprocessing protocols, such as data transformations and gene selections, making it difficult to use a universally applicable metric to fairly evaluate and benchmark all methods. To compare models' performance in predicting cell-type-specific GEPs in a way that is relatively fair to the method itself and across models, we calculated the metric scores (CCC and L1) in slightly different ways for each method. Details are listed below.

For TAPE: after log transformation, each sample is further transformed under MinMaxScaler for both simulated pseudobulk samples and real bulk samples. The benchmarking metrics are only available in the space after log2 and MinMax transformation. This is not a strictly fair comparison with other models, since the TAPE output values all range from 0 to 1 after MinMax transformation, resulting in a small L1 error. We still include the numbers in this table because this is the only valid way to evaluate TAPE.

For BayesPrism: the algorithm takes raw GEPs as input, and our evaluation is performed between BayesPrism's output and the raw cell-type-specific GEPs without any transformation. This comparison is the most favorable to BayesPrism; in other words, it outputs the highest correlation value among the different evaluation methods (i.e., comparisons against library-size and log-transformed GEPs). The caveat is that this comparison would yield large L1 error values. Also, BayesPrism does not allow a customized gene list as output when predicting cell-type-specific GEPs. For PBMC, we input the whole transcriptome into the algorithm following the instructions, and BayesPrism output 2772 genes in the prediction. Only 491/3004 overlapped with our training gene list and were included in the calculation of the CCC and L1 metrics. For the case of pancreatic, we input the whole transcriptome into the algorithm following the instructions, and BayesPrism output 14529 genes. 2226/2489 overlapped with our training gene list and were included in the metric calculations.

For CIBERSORTx: We compare CIBERSORTx's output against the log-transformed cell-type-specific GEPs.

Generalizability of BLUE

Generalizability is an important issue in algorithm development. Here, our simulation and training strategy is always generalizable, meaning that for different biological contexts (different tissues, different disease conditions, different cell types of interest, etc), our model can be retrained to adapt to the given biological context following the same simulation and training strategy we proposed in the paper. Within the same biological context, our trained model can be generalized to different datasets (from different sequencing platforms, different studies, etc), which has been demonstrated

in the experiments we presented in the paper (Table G). In these experiments, we have tested a wide range of sequencing platforms, tissue types, and health conditions to demonstrate the robustness and generalizability of our proposed deconvolution pipeline.

Cox model hazard ratios

We performed Cox proportional hazards regression to each TCGA AML patient groups following the same settings in the original AML paper [3] and plotted the confidence interval in Fig E. Our definition of favorable group (labeled as "favorable") is associated with 84% reduction in hazard with a 95% confidence interval (CI) [0.04, 0.66], while the GMP groups (the most favorable patient group defined in [3]) is associated with 59% reduction in hazard with a 95% CI [0.21, 0.78]. Our definition of unfavorable group (labeled as "unfavorable") is associated with 160% increase in hazard with a 95% CI [1.26, 5.37] while the Primitive groups (the most unfavorable patient group defined in [3]) is associated with a 6% increase in hazard with a 95% CI [0.64, 1.77].

After the multivariable adjustment, our definition of favorable and unfavorable group still exhibits strong association with the hazard comparing to previous definitions, as well as the other factors such as age, gender, cytogenetics. Although the "favorable" label in cytogenetic risk has a strong association with the reduction in hazard, the "poor" label in cytogenetic risk did not achieve a positive association with the increase in hazard, which demonstrates the existing survival factors might not be able to diagnose poor prognosis in AML.

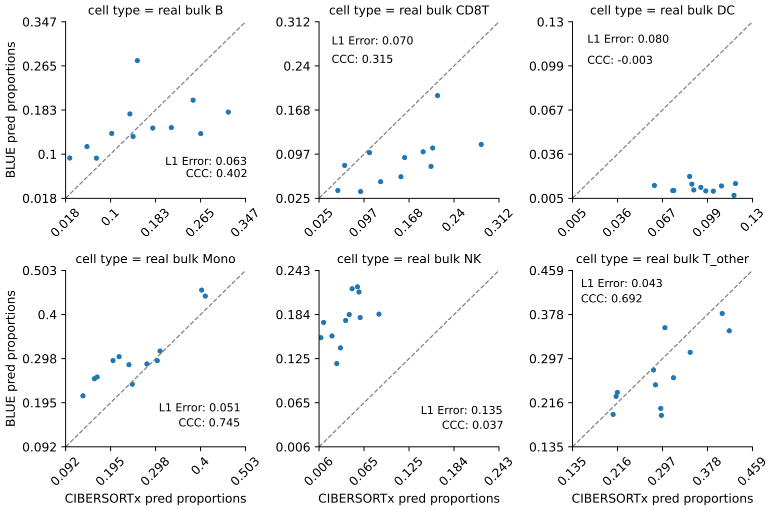

Fig. A Comparing predictions of cell-type proportions for real PBMC bulk samples: BLUE vs. CIBERSORTx.

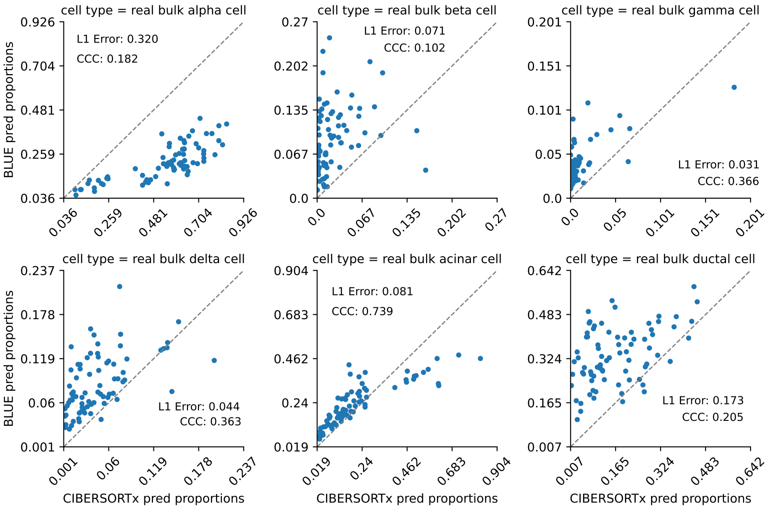

Fig. B Comparing predictions of cell-type proportions: BLUE vs. CIBERSORTx, in deconvolving real pancreatic islets bulk samples from study [1]. CIBERSORTx's prediction results are provided by MuSiC paper[2].

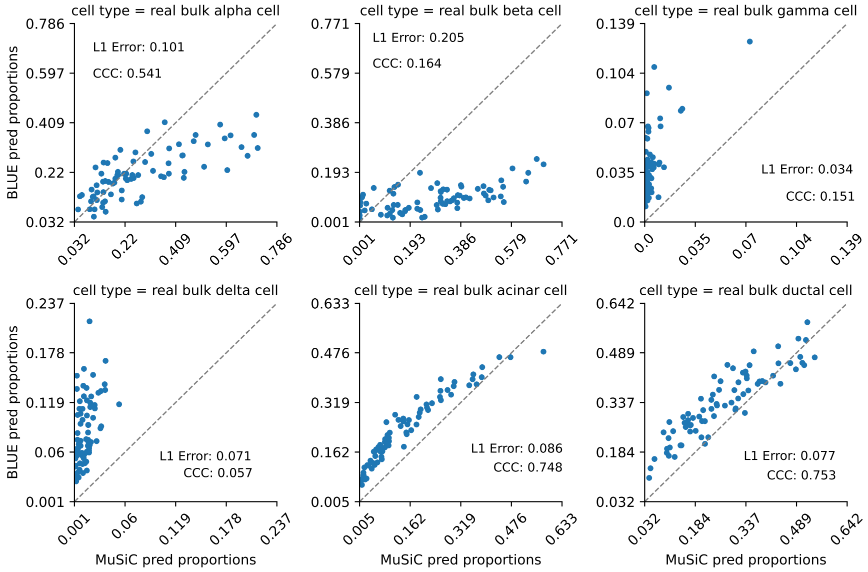

Fig. C Comparing predictions of cell-type proportions: BLUE vs. MuSiC, in deconvolving real pancreatic islets bulk samples from study [1]. MuSiC's prediction results are provided by MuSiC paper [2].

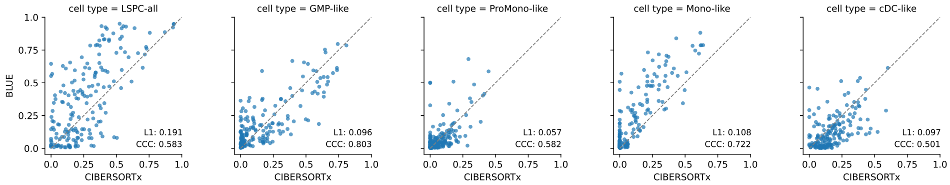

Fig. D Comparing predictions of cell-type proportions: BLUE vs. CIBERSORTx, in deconvolving real AML bulk samples from TCGA atlas. CIBERSORTx's prediction results are provided by [3].

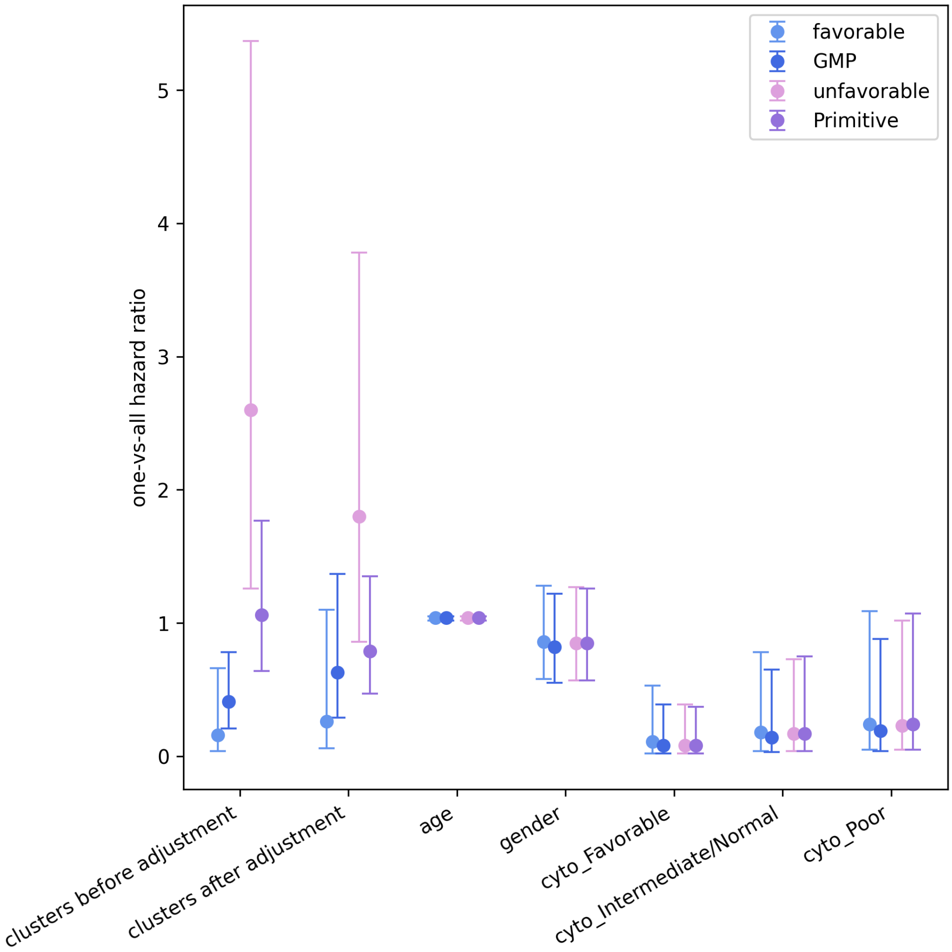

Fig. E Cox model hazard ratios for TCGA AML patient group definitions. The first column is the results before multivariable adjustment, and the remaining columns are the results after multivariable adjustment.

Table A PBMC cell-type proportions CCC(↑). "-" means the prediction is all 0 and CCC is not defined. **Bold** number is the best score and underlined number is the second best score in each column. The last line includes the standard deviation of BLUE's predictions across 3 experiments.

Cell type	B	CD8T	DC	Mono	NK	T other
Bisque	0.1098	-0.3637	0.0055	0.2863	-0.1557	-0.2098
SCDC	-	-	0.0023	0.1609	0.0115	-
DWLS	0.8092	0.4881	0.1970	0.5417	0.4687	**0.5038**
Scaden	0.6657	**0.5474**	0.0758	0.5514	0.4006	0.1643
MuSiC	-	-	-0.0013	0.3089	0.0086	-
CIBERSORTx	0.4119	0.5009	0.0019	0.0166	0.0601	0.0583
BayesPrism	**0.8955**	0.2750	0.0099	0.1337	0.3286	0.0865
TAPE	0.5199	0.2187	0.0272	0.6118	**0.4901**	0.0767
BLUE (μ)	0.4702	0.2238	**0.6658**	**0.6225**	0.3209	0.4145
BLUE ($\pm\sigma$)	0.4702 ± 0.0136	0.2238 ± 0.0222	0.6658 ± 0.0448	0.6225 ± 0.0742	0.3209 ± 0.0615	0.4145 ± 0.0405

Table B PBMC cell-type proportions L1(↓). The last line includes the standard deviation of BLUE's predictions across 3 experiments.

Cell type	B	CD8T	DC	Mono	NK	T other
Bisque	0.0496	0.0926	0.0228	0.0424	0.0755	0.0824
SCDC	-	-	0.0686	0.1592	0.3996	-
DWLS	0.0157	0.0756	0.0099	0.0576	0.0435	0.0640
Scaden	0.0325	0.0490	0.0170	0.0663	0.0512	0.1262
MuSiC	-	-	0.0678	0.0827	0.4771	-
CIBERSORTx	0.0529	0.0431	0.4555	0.1876	0.0674	0.2624
BayesPrism	0.0130	0.0830	0.0977	0.1780	0.0605	0.2245
TAPE	0.0419	0.0479	0.0248	0.0511	0.0391	0.1537
BLUE (μ)	0.0528	0.1043	0.0023	0.0532	0.0669	0.0658
BLUE ($\pm\sigma$)	0.0528 ± 0.0017	0.1043 ± 0.0050	0.0023 ± 0.0004	0.0532 ± 0.0092	0.0669 ± 0.0151	0.0658 ± 0.0085

Table C PBMC cell-type-specific GEPs CCC (↑). The last line includes the standard deviation of BLUE's predictions across 3 experiments.
[1] The predicted cell-type-specific GEPs of BayesPrism are compared with raw GEPs. Containing 483/3004 genes overlapped with the given gene list.
[2] The predicted cell-type-specific GEPs of TAPE are compared with ground truth cell-type-specific GEPs after log2 and MinMaxScaler transformation for each sample.

			**Sample 925L**			
Cell type	B	CD8T	DC	Mono	NK	T other
CIBERSORTx	0.0373	0.1523	0.3220	0.0960	0.0000	0.0000
BayesPrism[1]	0.4254	0.2404	0.1913	0.5176	0.2414	0.1628
TAPE[2]	0.7757	0.0279	0.5045	0.6109	0.6529	0.4776
BLUE (μ)	0.8037	0.8087	0.8334	0.8530	0.8051	0.8037
BLUE ($\pm\sigma$)	0.8037 ± 0.0137	0.8087 ± 0.0073	0.8334 ± 0.0062	0.8530 ± 0.0033	0.8051 ± 0.0041	0.8037 ± 0.0023
			**Sample 9JD4**			
Cell type	B	CD8T	DC	Mono	NK	T other
CIBERSORTx	0.0373	0.1495	0.3034	0.0849	0.0000	0.0000
BayesPrism[1]	0.3091	0.2346	0.1836	0.4322	0.2530	0.3340
TAPE[2]	0.7162	0.0246	0.5026	0.7810	0.6308	0.5551
BLUE (μ)	0.7779	0.8077	0.8287	0.8505	0.8332	0.8103
BLUE ($\pm\sigma$)	0.7779 ± 0.0159	0.8077 ± 0.0095	0.8287 ± 0.0067	0.8505 ± 0.0026	0.8332 ± 0.0035	0.8103 ± 0.0026
			**Sample G4YW**			
Cell type	B	CD8T	DC	Mono	NK	T other
CIBERSORTx	0.0255	0.1579	0.3112	0.1011	0.0000	0.0000
BayesPrism[1]	0.2684	0.1249	0.1863	0.6098	0.2970	0.3016
TAPE[2]	0.7087	0.0272	0.4996	0.6922	0.6939	0.5037
BLUE (μ)	0.7795	0.8000	0.8150	0.8684	0.8467	0.8218
BLUE ($\pm\sigma$)	0.7795 ± 0.0141	0.8000 ± 0.0092	0.8150 ± 0.0065	0.8684 ± 0.0039	0.8467 ± 0.0027	0.8218 ± 0.0023

Table D PBMC cell-type-specific GEPs L1 (↓). The last line includes the standard deviation of BLUE's predictions across 3 experiments.
[1] The predicted cell-type-specific GEPs of BayesPrism are compared with raw GEPs. Containing 483/3004 genes overlapped with the given gene list. BayesPrism does not allow a customized gene list when predicting cell-type-specific GEPs.
[2] The predicted cell-type-specific GEPs of TAPE are compared with ground truth cell-type-specific GEPs after MinMaxScaler transformation for each sample.

			**Sample 925L**			
Cell type	B	CD8T	DC	Mono	NK	T other
CIBERSORTx	5.0067	4.1546	3.6546	3.7280	3.0090	3.1217
BayesPrism[1]	66.9621	77.0030	153.5139	120.7639	78.9811	80.4979
TAPE[2]	0.0954	0.3415	0.2216	0.1721	0.1353	0.2414
BLUE (μ)	1.5024	1.6316	1.3916	1.2917	1.5977	1.6193
BLUE ($\pm\sigma$)	1.5024 ± 0.0739	1.6316 ± 0.0379	1.3916 ± 0.0325	1.2917 ± 0.0204	1.5977 ± 0.0195	1.6193 ± 0.0135
			**Sample 9JD4**			
Cell type	B	CD8T	DC	Mono	NK	T other
CIBERSORTx	5.0418	4.1828	3.7431	3.7608	3.1295	3.0893
BayesPrism[1]	68.4105	79.3375	155.9375	132.5445	84.3560	72.3243
TAPE[2]	0.1057	0.3400	0.2174	0.1102	0.1442	0.2025
BLUE (μ)	1.6590	1.6299	1.4075	1.3120	1.4827	1.5870
BLUE ($\pm\sigma$)	1.6590 ± 0.0842	1.6299 ± 0.0506	1.4075 ± 0.0339	1.3120 ± 0.0165	1.4827 ± 0.0186	1.5870 ± 0.0153
			**Sample G4YW**			
Cell type	B	CD8T	DC	Mono	NK	T other
CIBERSORTx	5.0679	4.1201	3.7072	3.7422	3.1224	3.1475
BayesPrism[1]	79.9260	80.7821	147.2980	118.7500	81.0929	69.1666
TAPE[2]	0.1059	0.3326	0.2182	0.1413	0.1292	0.2356
BLUE (μ)	1.6292	1.6561	1.4847	1.2371	1.4007	1.5100
BLUE ($\pm\sigma$)	1.6292 ± 0.0755	1.6561 ± 0.0528	1.4847 ± 0.0316	1.2371 ± 0.0229	1.4007 ± 0.0180	1.5100 ± 0.0120

Table E Pancreatic islets cell-type-specific GEPs CCC (↑). The last line includes the standard deviation of BLUE's predictions across 3 experiments.
[1] The predicted cell-type-specific GEPs of BayesPrism are compared with raw GEPs. Containing 2226/2489 genes overlapped with the given gene list. BayesPrism does not allow a customized gene list when predicting cell-type-specific GEPs.
[2] The predicted cell-type-specific GEPs of TAPE are compared with ground truth cell-type-specific GEPs after log2 and MinMaxScaler transformation for each sample.
* CIBERSORTx output all "1"s as the prediction for cell-type-specific GEPs.

			**Sample H3**		
Cell type	alpha	beta	gamma	acinar	ductal
BayesPrism[1]	0.1250	0.9450	0.2847	0.2291	0.0553
TAPE[2]	0.0856	0.4052	0.1145	0.2773	0.2818
BLUE (μ)	0.8974	0.8849	0.8974	0.9306	0.9325
BLUE ($\pm\sigma$)	0.8974 ± 0.0014	0.8849 ± 0.0057	0.8974 ± 0.0005	0.9306 ± 0.0017	0.9325 ± 0.0008
			**Sample H4**		
Cell type	alpha	beta	gamma	acinar	ductal
BayesPrism[1]	0.5210	0.7358	0.4260	0.0161	0.0318
TAPE[2]	0.1452	0.4496	0.0744	0.1853	0.2607
BLUE (μ)	0.9615	0.8644	0.7806	0.8591	0.9369
BLUE ($\pm\sigma$)	0.9615 ± 0.0002	0.8644 ± 0.0044	0.7806 ± 0.0019	0.8591 ± 0.0120	0.9369 ± 0.0016
			**Sample H6**		
Cell type	alpha	beta	gamma	acinar	ductal
BayesPrism[1]	0.2821	0.3815	0.4472	0.0223	0.0167
TAPE[2]	0.1119	0.4823	0.1143	0.1571	0.2138
BLUE (μ)	0.9395	0.8736	0.9142	0.7767	0.8255
BLUE ($\pm\sigma$)	0.9395 ± 0.0003	0.8736 ± 0.0002	0.9142 ± 0.0006	0.7767 ± 0.0100	0.8255 ± 0.0176
			**Sample T2D1**		
Cell type	alpha	beta	gamma	acinar	ductal
BayesPrism[1]	0.3705	0.8567	0.8320	0.0485	0.0601
TAPE[2]	0.1290	0.4235	0.1065	0.1806	0.2242
BLUE (μ)	0.9595	0.8895	0.9436	0.9156	0.8137
BLUE ($\pm\sigma$)	0.9595 ± 0.0002	0.8895 ± 0.0048	0.9436 ± 0.0009	0.9156 ± 0.0021	0.8137 ± 0.0091
			**Sample T2D2**		
Cell type	alpha	beta	gamma	acinar	ductal
BayesPrism[1]	0.4319	0.4820	0.1833	0.1231	0.2171
TAPE[2]	0.1387	0.3957	0.1238	0.1728	0.2925
BLUE (μ)	0.9556	0.9019	0.8839	0.9297	0.9465
BLUE ($\pm\sigma$)	0.9556 ± 0.0005	0.9019 ± 0.0006	0.8839 ± 0.0005	0.9297 ± 0.0016	0.9465 ± 0.0033

Table F Pancreatic islets cell-type-specific GEPs L1 (↓). The last line includes the standard deviation of BLUE's predictions across 3 experiments.
[1] The predicted cell-type-specific GEPs of BayesPrism are compared with raw GEPs. Containing 2226/2489 genes overlapped with the given gene list. BayesPrism does not allow a customized gene list when predicting cell-type-specific GEPs.
[2] The predicted cell-type-specific GEPs of TAPE are compared with ground truth cell-type-specific GEPs after log2 and MinMaxScaler transformation for each sample.
* CIBERSORTx output all "1"s as the prediction for cell-type-specific GEPs.

			**Sample H3**		
Cell type	alpha	beta	gamma	acinar	ductal
BayesPrism[1]	58.1781	20.3282	36.7909	172.5576	97.3916
TAPE[2]	0.2198	0.2224	0.5409	0.1493	0.4131
BLUE (μ)	0.9498	1.0039	0.8925	0.7730	0.8971
BLUE ($\pm\sigma$)	0.9498 ± 0.0054	1.0039 ± 0.0423	0.8925 ± 0.0020	0.7730 ± 0.0228	0.8971 ± 0.0068
			**Sample H4**		
Cell type	alpha	beta	gamma	acinar	ductal
BayesPrism[1]	40.9665	15.5875	20.6338	204.2021	121.3133
TAPE[2]	0.1738	0.1693	0.5946	0.1649	0.4334
BLUE (μ)	0.5812	1.0159	1.2126	1.1049	0.8912
BLUE ($\pm\sigma$)	0.5812 ± 0.0028	1.0159 ± 0.0263	1.2126 ± 0.0068	1.1049 ± 0.0870	0.8912 ± 0.0165
			**Sample H6**		
Cell type	alpha	beta	gamma	acinar	ductal
BayesPrism[1]	50.0074	46.8851	36.1350	174.1158	112.6091
TAPE[2]	0.1784	0.1808	0.5494	0.1698	0.4678
BLUE (μ)	0.7139	1.0650	0.8062	1.4887	1.5691
BLUE ($\pm\sigma$)	0.7139 ± 0.0025	1.0650 ± 0.0040	0.8062 ± 0.0017	1.4887 ± 0.0617	1.5691 ± 0.1056
			**Sample T2D1**		
Cell type	alpha	beta	gamma	acinar	ductal
BayesPrism[1]	47.7190	22.9775	19.7794	141.9842	91.4512
TAPE[2]	0.1898	0.2027	0.5498	0.1805	0.4589
BLUE (μ)	0.5784	0.9606	0.6617	0.8839	1.5826
BLUE ($\pm\sigma$)	0.5784 ± 0.0009	0.9606 ± 0.0380	0.6617 ± 0.0098	0.8839 ± 0.0098	1.5826 ± 0.0544
			**Sample T2D2**		
Cell type	alpha	beta	gamma	acinar	ductal
BayesPrism[1]	46.5889	42.5403	52.5754	158.6016	101.8921
TAPE[2]	0.1827	0.2463	0.5376	0.1870	0.4111
BLUE (μ)	0.6240	0.9445	0.9723	0.8190	0.7930
BLUE ($\pm\sigma$)	0.6240 ± 0.0041	0.9445 ± 0.0169	0.9723 ± 0.0037	0.8190 ± 0.0063	0.7930 ± 0.0295

Table G Summary of sequencing platforms and data source.

Biological context	PBMC	Pancreatic Islets	AML
Training data	scRNA-seq datasets profiled by 10x Genomics	scRNA-seq data generated by the Smart-seq2 protocol and sequenced by Illumina HiSeq 2000	scRNA-seq data profiled by short-read Illumina sequencing
Test data 1	an independent scRNA-seq cohort profiled by 10x Genomics	Bulk RNA-seq data profiled by NextSeq 500	TCGA: bulk RNA-seq data profiled by RNA-seq Illumina GA
Test data 2	real bulk RNA-seq samples profiled by Illumina HiSeq 2000	-	TARGET: bulk RNA-seq data profiled by Affymetrix Gene ST Array

References

[1] Fadista J, Vikman P, Laakso EO, et al (2014) Global genomic and transcriptomic analysis of human pancreatic islets reveals novel genes influencing glucose metabolism. Proceedings of the National Academy of Sciences 111(38):13924–13929

[2] Wang X, Park J, Susztak K, et al (2019) Bulk tissue cell type deconvolution with multi-subject single-cell expression reference. Nature communications 10(1):380

[3] Zeng AG, Bansal S, Jin L, et al (2022) A cellular hierarchy framework for understanding heterogeneity and predicting drug response in acute myeloid leukemia. Nature medicine 28(6):1212–1223
